# Blood feeding patterns of mosquitoes: random or structured?

**DOI:** 10.1186/1742-9994-7-3

**Published:** 2010-01-21

**Authors:** Luis F Chaves, Laura C Harrington, Carolyn L Keogh, Andy M Nguyen, Uriel D Kitron

**Affiliations:** 1 Department of Environmental Studies, Emory University, Atlanta GA 30322, USA; 2 Department of Entomology, Cornell University, Ithaca NY 14853, USA; 3 Odum School of Ecology, University of Georgia, Athens GA 30602, USA; 4 Department of Epidemiology, Mailman School of Public Health, Columbia University, New York NY 10032, USA

## Abstract

**Background:**

The foraging behavior of blood-sucking arthropods is the defining biological event shaping the transmission cycle of vector-borne parasites. It is also a phenomenon that pertains to the realm of community ecology, since blood-feeding patterns of vectors can occur across a community of vertebrate hosts. Although great advances in knowledge of the genetic basis for blood-feeding choices have been reported for selected vector species, little is known about the role of community composition of vertebrate hosts in determining such patterns.

**Methods & Results:**

Here, we present an analysis of feeding patterns of vectors across a variety of locations, looking at foraging patterns of communities of mosquitoes, across communities of hosts primarily comprised of mammals and birds. Using null models of species co-occurrence, which do not require ancillary information about host abundance, we found that blood-feeding patterns were aggregated in studies from multiple sites, but random in studies from a single site. This combination of results supports the idea that mosquito species in a community may rely primarily on host availability in a given landscape, and that contacts with specific hosts will be influenced more by the presence/absence of hosts than by innate mosquito choices. This observation stresses the importance of blood-feeding plasticity as a key trait explaining the emergence of many zoonotic mosquito transmitted diseases.

**Discussion:**

From an epidemiological perspective our observations support the idea that phenomena promoting synchronization of vectors and hosts can promote the emergence of vector-borne zoonotic diseases, as suggested by observations on the linkages between deforestation and the emergence of several human diseases.

## Background

One of the goals of evolutionary ecology is to understand the ecological niche of species as a product of changing environments and their evolutionary background [1,2]. Patterns of similarity in resource use across species in a community led to the proposal of guilds as a conceptualization of the interactions of species within the same trophic level [3]. One of the ways to understand patterns of resource use by consumers belonging to a given guild is to assume that genetic components influence feeding preferences, as proposed for mosquitoes [4,5]. This approach is based in a conservative view of niches, the notion that the evolutionary history of a consumer taxon limits the resources it exploits [6,7]. However, as thoroughly accounted by Southwood [8], the niche of a species is primarily shaped by the environment where its life history takes place. As shown by MacArthur [9], related species can forage across all the available and suitable resources, but will tend to specialize in space or time to forage, thus rendering co-existence possible through niche partitioning. Although well grounded in field observations, MacArthur's conclusions came from the observation of a group of organisms, warblers, whose life history keeps the same niche through development. However, a wide variety of organisms have niche shifts during their ontogenetic development, and the adaptive usage of different resources during the different life history stages can lead to ecological patterns that deviate from the expectation of boundaries in resource use. Mosquitoes are one of many groups that have major shifts in resource exploitation directly related to their holometabolism, by which they change from aquatic (pre-adult stages) to terrestrial (adult) habitats; they are of particular importance for their role as both vectors of pathogens and nuisance [10].

It is well known that for aquatic mosquito larvae, spatio-temporal heterogeneities in larval habitats play a key role in shaping the community structure [11,12], with different degrees of co-existence regulated by the dynamics of the external environment, e.g., climatic variability [13-15]. Co-existence of mosquito species larvae is also supported by differences in traits related to foraging on different resources [16]. However, for adults, mouthparts for blood sucking are similar enough to enable blood-feeding foraging across most vertebrate hosts [17,18]. Thus, the extent to which blood-feeding patterns of mosquitoes are shaped by the availability of blood-feeding resources, i.e., vertebrate hosts, versus innate feeding choice specificity, is a relevant question. From an applied perspective, understanding adult mosquito blood-foraging patterns on a community of hosts is essential for the identification of species that could be involved in the transmission of vector-borne pathogens in nature (see Additional file 1: *Biodiversity and zooprophylactic effects*). In principle, zooprophylaxis has the potential to reduce human risk for vector-borne pathogen transmission in settings with increased vertebrate biodiversity (see Additional file 2: *Zooprophylaxis and dilution effect: the same natural history seen with different glasses*). Identification of reservoirs/amplifying hosts also can guide effective targeting of species to be managed (by culling or vaccination) for disease control [19].

Mosquito blood foraging can be studied by direct observation [20,21], the use of baited traps [22] or by the analysis of blood contents in the mosquito gut [23]. Blood in a mosquito gut can be identified using a range of tools, which have evolved through time. These approaches have benefited from advances in the fields of immunology and molecular biology. From techniques based on immunological reactions [24,25] to PCR-based DNA identification [26,27] mosquito feeding patterns can be tested against a set of suspected hosts and across different landscapes (see Additional file 3: *Mosquito blood-feeding analysis: from precipitin tests to DNA fingerprinting*). Traditional approaches to analyzing host choice by vectors include the computation of the host utilization rate [28,29], which is the percent of bloodmeals belonging to a focal host species (used as a proportion in models); the forage ratio [29] which is the host utilization rate divided by the proportional abundance of the focal host species; and the feeding index[30] which is defined as the proportion of feeds on one host with respect to another divided by the expected proportion of feeds on these two hosts based on factors affecting feeding, such as host abundance and size. Some of these indices require information on the composition of the vertebrate host community which is often very difficult to gather. The lack of information on host community composition renders impossible the testing for randomness in blood-feeding patterns of adult mosquitoes. However, the qualitative information produced by patterns of presence/absence of specific host utilization across different mosquito species in a given setting, or across habitats for a given mosquito species, can be used to study feeding patterns without information on the host community composition, since resulting patterns are similar to those studied in community ecology, especially those used to study rules of community assemblage [31]. Specifically, the number of species pairs that never co-occur, or 'checkerboards' [32], can be used to test hypotheses about segregation in the blood-feeding patterns of a community of vectors through the use of null-model tests. The Additional file 4: *A Primer of null model testing of blood-feeding patterns *illustrates the logic behind null model testing of vector blood-feeding patterns.

Null-model tests are tools originally proposed to test the randomness in community assemblages [33]. Nowadays, null-models are considered pattern-generating models based on randomization of ecological data or on random sampling from a known or arbitrary distribution [34]. Here, we analyze presence/absence patterns using data from published studies on mosquito blood-feeding patterns. Null-models are used to test whether mosquito foraging occurs randomly, i.e., revealing no host-specificity, or if mosquitoes obtain bloodmeals only from certain host species, either with a strong segregation of host species choice or the aggregation around at least one host. Each hypothesis can be respectively supported by random, segregated patterns (some host species fed upon only by some mosquito species), and aggregated (same host species fed upon by all mosquito species).

## Methods

We searched PubMed http://www.ncbi.nlm.nih.gov (1966 to October 2008), ISI Web of Science http://www.isiknowledge.com (1916 to October 2008), the American Journal of Tropical Medicine and Hygiene, AJTMH http://www.ajtmh.org (1921 to February 2009) and Journal of Medical Entomology, JME (1964 to January 2009) to identify field studies on blood-feeding patterns of mosquito communities across a range of hosts. We used the following key-words for our search: *mosquito *and *blood *or *host *and *feeding *or *foraging*. We did not search in journals not indexed in the above referred databases and limited our search to articles in English, French, Spanish, Portuguese, Italian, Vietnamese and Hebrew, the languages dominated by the authors. We discarded articles that were focused on only one mosquito species, studies that were focused on multiple bloodmeals without reference to the ecological context of mosquito sampling and studies where mosquito collection was done with cue-specific baits, i.e., animals. We kept studies showing results on the feeding patterns of a community of mosquito species on a community of vertebrate hosts, where samples came from resting boxes, aspirators, gravid and light traps, i.e., host-independent sampling methods. We did a more thorough search in the AJTMH and JME after realizing that most of the articles with all conditions for inclusion in our study came from these two journals. Some of the manuscripts had unidentified bloodmeal sources that were ignored for the analyses. We focused on mosquito communities because only at this level of complexity can comparisons be made in order to determine if there is a strong segregation (segregated pattern) of host species by vector species that could forage across a community of vertebrate hosts. Studies were also characterized as single or multiple site, depending on the adjacency of sampling sites.

Null-model analyses were performed using the C-score [35,36]. The best alternative is the number of checkerboards, which is the count of pairs of species that never co-occur (feed) in an ensemble of host species [32]. However, in some cases mosquito species can have some overlap in their feeding patterns though they share such overlap only for a small subset of potential hosts. To overcome this limitation the C-score was proposed, which is computed by the following equation:(1)

Where, for the case of our analysis *S*_*i *_and *S*_*j *_are the total number of host species for mosquito species *i *and *j*, *Q*_*ij *_the number of times mosquito species *i *and *j *share a host, and *R *the total number of mosquito species. This metric measures species segregation but does not require perfect checkerboards [36]. An additional reason to choose the C-score is the superior statistical qualities of this index that make it a more reliable indicator of co-occurrence patterns when compared with less flexible indexes like the checkerboards [34]. The tools described here are also useful for studying patterns of blood feeding for individual species across habitats and hosts.

For the datasets we tested the null hypothesis that mosquito species are equally likely to forage on any host species. The null models were fixed-equiprobable, i.e., row sums of the original matrix are preserved and column values are the same. The latter means the following in the context of this research: when a new random dataset is generated, the probability of a mosquito species (rows) feeding on any of the hosts (columns) is the same, but the number of host species upon which it can feed is proportional to the number of host species on which it fed in the original dataset. Once the output of all simulations is obtained, which are the C-scores for each simulated dataset, a distribution is constructed and the significance of the C-score obtained for the original dataset is compared to this distribution of simulated indices, and inference is based on how extreme is the C-score calculated from the original dataset when compared to the distribution of simulated C-scores. When the C-score obtained from the data is statistically significantly larger than that of the simulations, it means that mosquito species have strict host-specific feeding (segregated) patterns; when it is not statistically different, it implies that feeding patterns are random, and when the C-score from the data is statistically significantly smaller than that from the simulations, it means that all mosquito species share at least one host species in their bloodmeals from the community of hosts, indicating an aggregated feeding pattern. We chose the fixed-equiprobable algorithm, because it provides a robust predictor [34,35], and allows to test the null hypothesis of equal preference among host species. Simulations were carried out using the software Ecosim 7.0 [34]. For the tests we used 5000 randomizations.

## Results

We found a total of 48 articles related to blood-feeding patterns in mosquitoes. A total of 19 articles had data on mosquito and vertebrate host communities, but only 12 fulfilled the criteria to be analyzed, since only these articles reported the blood contents of all engorged mosquito species studied (Table 1). We noticed that studies from the states of Florida [37-42] and Connecticut [43,44] in the USA, sampled a geographically restricted set of sites, with similar communities of hosts, but reported mosquito host use for selected species separately. To investigate the impacts of these potential biases on the analyses, we performed individual analyses for both the individual reports and for the full datasets obtained by joining the data from all the different mosquito and host species reported for each state (i.e., Florida and Connecticut), raising the total number of analyzed studies to 14 (Table 1). More than 75% of the studies were done in the US. The regional bias of our study reflects the fact that most blood-feeding studies have tended to be narrowly focused on "main vectors". For example, several studies on blood feeding patterns have been carried out in Africa, Asia and Latin America, but only one from the latter fulfilled the selection criteria of including the vector community (Table 1), while the rest were focused on the "main vector". The most common mammal host species (Additional file 5: Table S1) were rabbits (81% of the studies with data on mammals), humans (75%) and horses (68%). For birds the most common across all studies specific to avian species (Additional file 6: Table S2) were: American Robin (100%), Gray Catbird, Northern Cardinal and Brown-headed Cowbird (each present in 83% of the studies). The most common mosquito species (Table 2) were *Culex pipiens *complex (47.37%), *Aedes vexans *(36.84%) and *Anopheles quadrimaculatus *(36.84%). We also found a great heterogeneity in the length of studies, ranging from a couple of weeks [26,45], to seasonal (covering one year of mosquito season [46]), to longitudinal (spanning in some case as much as at least five years [37-42]), see Additional file 7: Table S3 for details on sampling frequency and study length.

**Table 1:** Selected studies, mosquito richness (number of mosquito species), vertebrate classes (Mammal = M, Amphibian = Am, Reptile = R, Avian = Av), location, number of sampling sites (Site) and collection method

Study	Mosquito richness (see Location)	Class (number of species)	Location(s)	Site	Collection method
Edman [37]	13	M(6), Am/R(1), Av(1)	Florida Marsh, USA	Single	Vehicle mounted aspirator
Apperson et al [26]	9	M(6), R(1), Av(1),	Borough of Queens, New York city, USA	Single	Backpack aspirator & hand held aspirators
Burkett-Cadena et al [91]	11	M, Am, R, Av*	Tuskegee National Forest, Alabama, USA	Single	CDC Light traps and vacuum aspirator
Nasci & Edman [92]	6	M (6)	Red maple and white cedar freshwater swamp, Massachusetts, USA	Single	Resting boxes
Forattini et al [46]	10	M(5), Am*, Av*	Forest, houses in several locations in Sao Paulo state, Brazil	Multiple	Aspirator & sweepers
Apperson et al [93]	11 (NJ), 9(NY)	M(10), Am*, R*, Av(18)	Several locations in the states of New York (NY) and New Jersey (NJ), USA	Multiple	Resting boxes
Savage et al [94]	10	M(9), Am*, R*, Av(24)	Several locations in Memphis and Shelby county, Tennessee, USA	Multiple	Aspirator
Molaei et al [44]	23	M(13), Am*, R*, Av(12)	Several locations in the state of Connecticut, USA	Multiple	CO_2_-baited CDC Light Trap, CDC gravid trap
Molaei et al [43]	3	M (11), Av(35)	31 locations in 6 counties of Connecticut, USA	Multiple	CO_2_-baited CDC Light Trap, CDC gravid trap, mosquito magnet trap
Hamer et al [47]	9	M(8), Am*, Av(33)	26 locations in suburban Chicago, USA	Multiple	CO_2_-baited CDC Light Trap, CDC gravid trap, Backpack aspirator
Kay et al [23]	10	M(7), Av(1)	9 locations in urban/suburban Brisbane, Australia	Multiple	CO_2_-baited CDC Light Trap
Fyodorova et al [45]	10(u), 7(r), 6(f)	M(7), Av(1)	Several locations: urban (u), rural(r), livestock farms(f) in/around Volgograd, Russia	Multiple	Backpack aspirator
Connecticut [43,44]	26	M(13), Am*, R*, Av(37)	31 locations in 6 counties of Connecticut, USA	Multiple	CO_2_-baited CDC Light Trap, CDC gravid trap, mosquito magnet trap
Florida [37-42]	31	M(9), Am*, R(3), Av(1)	Several locations in Florida, USA	Multiple	Vehicle mounted aspirator; Resting boxes, light trap Collections

* Only presented as Class

**Table 2:** Mosquito communities (species recorded in the studies)

Study	Bloodfed Species
**Florida **[37-42], *indicates Edman [37] Table 6, Area 2	Aedes atlanticus *, Aedes fulvus pallens*, Aedes infirmatus *, Aedes mitchellae, Aedes sollicitans, Aedes taeniorhynchus *, Aedes triseriatus, Aedes vexans*, Anopheles crucians *, Anopheles quadrimaculatus *, Culex erraticus, Culex iolambdis, Culex opisthopus, Culex peccator, Culex pilosus, Culex quinquefasciatus, Culex salinarius, Culex territans, Culex restuans, Culex nigripalpus, Culiseta melanura, Culiseta inornata, Coquillettidia perturbans*, Deinocerites cancer, Mansonia titillans*, Psorophora ciliata *, Psorophora confinnis*, Psorophora ferox *, Psorophora howardii*, Wyeomyia vanduzeei, Wyeomyia mitchellii
**Connecticut **[43,44], * indicates Molaei et al [43]	Aedes abserratus, Aedes aurifer, Aedes canadensis, Aedes cantator, Aedes cinereus, Aedes communis, Aedes excrucians, Aedes japonicus, Aedes sollicitans, Aedes sticticus, Aedes stimulans, Aedes taeniorhynchus, Aedes thibaulti, Aedes triseriatus, Aedes trivittatus, Anopheles barberi, Anopheles punctipennis, Anopheles quadrimaculatus, Anopheles walkeri, Coquilletidia perturbans, Culex territans, Culex pipiens *, Culex restuans *, Culex salinarius *, Psorophora ferox, Uranotaenia sapphirina
Apperson et al [26]	*Aedes cinereus, Aedes vexans, Ochlerotatus cantator, Ochlerotatus sollicitans, Ochlerotatus triseriatus, Coquillettidia perturbans, Culex salinarius, Culex restuans, Culex pipiens*
Burkett-Cadena et al [91]	*Anopheles crucians, Anopheles punctipennis, Anopheles quadrimaculatus, Coquillettidia perturbans, Culiseta melanura, Culex erraticus, Culex peccator, Culex quinquefasciatus, Culex restuans, Culex territans, Ochlerotatus sticticus*
Nasci & Edman [92]	*Aedes abserratus, Aedes aurifer, Aedes canadensis, Aedes cantator, Aedes cinereus, Aedes vexans*
Forattini et al [46]	*Aedes scapularis, Aedes serratus, Anopheles cruzii, Coquillettidia chrysonotum, Coquillettidia venezuelensis, Coquillettidia coronator, Culex riberiensis, Culex sacchettae, Psorophora albigenus, Psorophora ferox*
Apperson et al [93]	New Jersey *Aedes vexans, Anopheles bradleyi, Anopheles crucians bradleyi, Anopheles punctipennis, Anopheles quadrimaculatus, Culiseta melanura, Culex pipiens, Culex restuans, Culex salinarius, Ochlerotatus sollicitans, Ochlerotatus thibaulti* New York *Aedes cinereus, Aedes vexans, Coquilletidia perturbans, Ochlerotatus canadensis, Ochlerotatus japonicus, Ochlerotatus taeniorhynchus, Ochlerotatus triseriatus, Ochlerotatus trivittatus, Psorophora ferox*
Savage et al [94]	*Anopheles punctipennis, Anopheles quadrimaculatus, Culex pipiens complex restuans, Culex pipiens complex, Culex pipiens pipiens, Culex pipiens quinquefasciatus, Culex pipiens quinquefaciatus hybrids, Culex restuans, Culex erraticus, Culex territans*
Hamer et al [47]	*Aedes vexans, Anopheles quadrimaculatus, Culex pipiens, Culex restuans, Culex salinarius, Culiseta inornata, Coquillettidia perturbans, Ochlerotatus triseriatus, Ochlerotatus trivittatus*
Kay et al [23]	*Aedes notoscriptus, Aedes procax, Aedes vigilax, Aedes vittiger, Culex annulirostris, Culex australicus, Culex quinquefasciatus, Culex sitiens, Coquillettidia linealis, Coquillettidia xanthogaster*
Fyodorova et al[45]	Urban *Anopheles claviger, Anopheles messeae, Aedes caspius, Aedes cinereus, Aedes vexans, Coquillettidia richiardii, Culex modestus, Culex pipiens pipiens, Culiseta annulata, Uranotaenia unguiculata* Rural *Anopheles messeae, Aedes caspius, Aedes flavescens, Aedes vexans, Culex modestus, Culex pipiens pipiens, Uranotaenia unguiculata* Livestock farms *Anopheles messeae, Aedes caspius, Aedes vexans, Coquillettidia richiardii, Culex modestus, Culex pipiens pipiens*

Table 3 shows a total of 12 studies with data on vertebrate classes. Table 4 presents 7 studies with data on avian species, and Table 5 has results for 11 studies on mammalian species. These three tables present the results of the C-score null model tests. At the vertebrate class level, mosquitoes fed in an aggregated pattern (Table 3), with most species feeding either on mammal or avian species (Additional files 5 and 6: Table S1 and Table S2). In general, studies from multiple locations had observations that correspond to larger spatial scales, e.g. counties and states, or metropolitan areas [47], when compared to single locations. The feeding patterns from studies from multiple sites were primarily aggregated.

**Table 3:** Patterns of vertebrate class host co-feeding.

Source	Site	C-score	Mean ± Variance	p < exp	p > exp	Pattern
Edman [37] Table 5	Single	0.058	0.478 ± 0.002	< 0.0001	1	Aggregate
Forattini et al [46] Table 3	Multiple	0.089	1.214 ± 0.0463	< 0.0001	1	Aggregate
Apperson et al [93] Table 1 (New York)	Multiple	0.967	0.858 ± 0.010	0.9332	0.0918	Random
Apperson et al [93] Table 1 (New Jersey)	Multiple	0.061	0.464 ± 0.009	0.0002	1	Aggregate
Savage et al [94] Table 1	Multiple	0.044	0.213 ± 0.004	0.0314	0.9816	Aggregate
Burkett-Cadena et al [91] Table 1	Single	0.509	0.526 ± 0.008	0.4196	0.6934	Random
Molaei et al [44] Table 1	Multiple	0.000	0.792 ± 0.003	< 0.0001	1	Aggregate
Hamer et al [47] Table 1	Multiple	0.000	0.415 ± 0.008	0.0006	1	Aggregate
Connecticut [43,44]	Multiple	0.000	0.848 ± 0.002	0	1	Aggregate
Florida [37-42]	Multiple	0.028	0.635 ± 0.001	< 0.0001	1	Aggregate

Source indicates the article and table within such article used for data extraction. Site describes single (adjacent) or multiple (non-adjacent) sampling sites in a given location. Values of the estimated C-score are the values calculated from the data, and Mean ± Variance are the results from the simulations. The values of p < exp and p > exp indicate the probability that the C-score value is significantly smaller (indicating aggregated pattern) or larger (segregated pattern) than that expected by random, with a p-value < 0.05 indicating statistical significance. When none of the p-values is below the threshold of 0.05 the pattern is random. Column "pattern" indicates the interpretation of the pattern.

**Table 4:** Patterns of bird species host co-feeding.

Source	Site	C-score	Mean ± Variance	p < exp	p > exp	Pattern
Apperson et al [26] Table 3	Single	5.000	4.775 ± 1.879	0.573	0.449	Random
Apperson et al [93] Table 3	Multiple	8.333	21.034 ± 1.391	0	1	Aggregate
Molaei et al [43] Table 3	Multiple	55.000	76.423 ± 70.769	0.003	0.999	Aggregate
Savage et al [94] Table 3	Multiple	25.611	53.021 ± 9.340	0	1	Aggregate
Molaei et al [44] Table 3	Multiple	2.694	3.704 ± 0.061	0.0016	0.998	Aggregate
Hamer et al [47] Table 2	Multiple	49.333	71.612 ± 64.190	0.0006	0.999	Aggregate
Connecticut [43,44]	Multiple	8.394	14.036 ± 1.697	0.0000	1	Aggregate

For heading explanations and interpretations of the p-values see Table 3.

**Table 5:** Patterns of mammal species host co-feeding.

Source	Site	C-score	Mean ± Variance	P < exp	p > exp	Pattern
Edman [37] Table 6, Area 2	Single	0.276	0.313 ± 0.001	0.163	0.853	Random
Nasci & Edman [92] Table 4	Single	2.067	3.092 ± 0.327	0.053	0.957	Random
Apperson et al [26] Table 2	Single	1.694	2.092 ± 0.083	0.101	0.912	Random
Apperson et al [93] Table 2 (New York)	Multiple	0.694	3.125 ± 0.032	0	1	Aggregate
Apperson et al [93] Table 2 (New Jersey)	Multiple	0.2	3.879 ± 0.173	0	1	Aggregate
Fyodorova et al [45] Table 4 (Livestock)	Multiple	0.466	2.581 ± 0.019	0	1	Aggregate
Fyodorova et al [45] Table 4 (Rural)	Multiple	0.867	4.0836 ± 0.3413	0	1	Aggregate
Fyodorova et al [45] Table 4 (Urban)	Multiple	1.6	2.401 ± 0.090	0.01	0.99	Aggregate
Kay et al [23] Table 1	Multiple	0.822	2.695 ± 0.127	0	1	Aggregate
Savage et al [94] Table 6	Multiple	1.444	2.812 ± 0.153	0.002	0.998	Aggregate
Molaei et al [44] Table 2	Multiple	1.340	4.847 ± 0.042	0	1	Aggregate
Hamer et al [47] Table 3	Multiple	3.000	13.138 ± 3.205	0	1	Aggregate
Connecticut [43,44]	Multiple	2.033	6.317 ± 0.060	0	1	Aggregate
Florida [37-42]	Multiple	1.069	3.813 ± 0.025	0	1	Aggregate

For heading explanations and interpretations of the p-values see Table 3.

For birds (Table 4), the patterns were also primarily aggregated. The only study that showed random patterns [26] was also at a very local scale from a single site in the city of New York. A similar pattern of aggregation and randomness was observed for mammals (Table 5) and differences in patterns inferred with the C-score and the number of checkerboards likely arose from the absence of perfect checkerboards.

In summary, all results indicated that feeding patterns for the mosquito species and localities studied were aggregated when multiple sites were sampled, but random across available choices at the single, probably more local spatial scale. The above generalizations are hold for the more comprehensive analysis of the mosquito faunas of: (i) Florida, where an increase in sampled locations led to an aggregate pattern as opposed to the random pattern observed at the single location; and (ii) Connecticut, where an increase in the richness of mosquito and host species led to a similar aggregated pattern as the one observed for the split mosquito community sampled across multiple locations.

## Discussion

Our results show that in studies where data were gathered from multiple sites, mosquito species showed an aggregated feeding pattern which indicated that most mosquito species shared at least one host species. This aggregated pattern could arise from host choice based on host availability as reported elsewhere [48,49]. For example, in the datasets we studied the most conspicuous example of aggregation around a given host was documented in Connecticut, USA [44], where all mosquito species fed on deer. Deer in New England, (which includes Connecticut) although not the most abundant vertebrate in terms of individuals, are a readily available source of blood to vectors [50]. The latter has been shown for adult ticks, with deer size and perhaps the lack of host defensive, anti-biting behavior, given as possible explanations [51]. Another complementary explanation may be the increased species richness in the meta-community of mosquitoes across multiple locations, as observed in Florida (Table S3), which is expected from the increase in the sampling of different mosquito habitats with multiple locations when compared to the coarser habitats of vertebrate hosts. For example, in Connecticut while deer are common in any natural area [44], the larval and resting habitats for different mosquito species are conditioned by vegetation and soil type [52].

By contrast to studies with multiple sites, studies with a single sampling location showed that feeding patterns did not deviate significantly from random, as can be expected given that restricted habitats offer limited resources [53]. The result of aggregation of bloodmeals in at least one vertebrate class or one host species by the community of mosquitoes is suggestive in the sense that it supports the critical role that synchronization in the encounter of host and blood-feeding insects plays in mosquito foraging [10]. This spatio-temporal co-occurrence is a more general requirement for any trophic interaction between a resource and a consumer [53]. This synchronization could also explain the absence of dilution effects in urban environments [54], since species also get more crowded in patches of habitat following habitat transformation and fragmentation [55], which are not always associated with biodiversity loss [56]. This finding contradicts the generalization that physiological factors are the major determinant of blood host choice by vectors, which postulates that close association with a narrow range of hosts would select for specialization in vector physiology that would limit the range of hosts that a species can exploit [18]. While this may be the case for fleas [57] and kissing bugs [58] it is important to note that these groups exploit a unique resource throughout their life histories, unlike female mosquitoes that shift from exploitation of aquatic resources, where they are often very specialized in terms of habitat choice [11], to blood feeding from terrestrial hosts.

In mosquitoes it has been argued that some species have strong predilection for specific bloodmeal sources, the best example being *Aedes aegypti *and its preference for humans [59,60]. However, blood-meal analyses for *Ae. aegypti *have shown that choices can be plastic, since bloodmeals can come from several species besides humans [60,61]. *Ae. albopictus*, another major dengue vector shows plasticity with regard to blood foraging: in Thailand, mosquitoes sampled from endemic dengue villages had fed exclusively on humans [60]; in North America where mosquito contact with humans is less likely to occur (given the generalized anti-mosquito screening of houses), this species was catholic in its feeding repertoire, with bloodmeals coming from very diverse sources, including several mammal and avian species [62,63]. In fact, a recent study found wildlife mammals infected with the dengue virus serotypes circulating in humans during several outbreaks in French Guiana, suggesting that dengue vectors feeding on infected humans can also feed on other mammal species [64]. Examples are not restricted to culicines. In fact, *Anopheles gambiae*, the major malaria vector that has been suggested to be highly anthropophilic [65] and does have a strong preference for humans even when given other choices of blood hosts under controlled field settings, uses cows as its primary blood source in areas of Burkina Faso where humans, because of the widespread use of bednets, are not available as blood source [66]. Thus, we propose that mosquito specialization in resource exploitation is likely to be primarily limited to pre-adult stages. Although differences in reproductive fitness of adult female mosquitoes fed over an array of potential hosts have been reported for some species [18], trade-offs in fitness performance may select for generalist behavior in blood-feeding patterns, or for alternative life history strategies, like autogeny, which is not uncommon in mosquitoes [49].

The randomness of host-feeding patterns by mosquitoes in single locations further reinforces the idea of non-specialization for blood sources in mosquitoes, especially when evaluated in conjunction with behavioral studies on host choices in the laboratory, and from field studies looking at seasonal changes in feeding patterns. Classical studies on mosquito blood-feeding behavior have shown that some mosquitoes can be opportunistic with regard to their feeding choices [67], and when differences have been observed they are related to the ease with which mosquitoes locate hosts [18,49,68], limitations in access to hosts [49,66], or by a non-heritable behavioral conditioning [69]. Patterns of blood host shift are also related to host abundance, as shown for *Culex nigripalpus *at subtropical latitudes [48], which fed on birds when they were the more abundant vertebrate. Aside from the effect of relative host abundance, the other major factor that can condition mosquito blood foraging is the defensive behavior of hosts [70-72]. Studies have found that defensive behavior can explain site selection for bites on a given host [73], and that differences in this behavior can be related to host age [73,74] and size [20,75]. The use of mechanical barriers such as bed nets [66,76] also influence mosquito blood foraging, as can health or stress effects related to pathogen infection in the host [77]. Thus, although more studies are required, especially of mosquito communities that comprise major vectors assumed to be highly anthropophilic, behavioral and environmental factors that are independent of genetic make-up could regulate mosquito blood foraging across a community of vertebrate hosts.

### Directions for future research and implications

Our study has some limitations related to the scarce knowledge about mosquito community ecology, and heterogeneities in the sampling and design of the studies we analyzed and the analytical tool we used to study the feeding patterns: a) the variable sampling techniques for the collection of blood-fed mosquitoes(whose effects on biasing mosquito species composition can be very variable[37-42,71]); b) the techniques for bloodmeal identification changed through time (following the development of molecular techniques); c) studies were geographically biased for North America; d) Lack of definition and quantification of the areas sampled (which precluded a formal definition of spatial scale); e) lack of definition and quantification of sample diversity to assess whether blood-fed mosquito species were representative of the whole community (we relied on the expert opinion expressed in the studies we analyzed) f) probabilities for host choice in the randomization algorithm were not weighted by the number of mosquitoes captured within a study that shared a given blood-meal type or by the abundance or biomass of potential hosts species. All these issues can be addressed as new studies focus in communities of mosquitoes, not only specific vectors. Also, it is necessary that ecological studies on mosquitoes more routinely incorporate the evaluation of species richness with well established quantitative methods, such as the extrapolation from species accumulation with sampling effort [78] and leave behind expert opinion as a criterion to support a representative sampling of mosquito diversity. Weighing can be easily implemented in the randomization algorithm of the null model tests, and very likely molecular tools for blood feeding analysis will evolve well beyond what is currently acknowledged as the "cutting edge".

Mosquito blood-feeding also has an additional layer of complexity, which is added by vector foraging heterogeneity on hosts belonging to the same species. For example, in a study of *Ae. albopictus*, 80% of human bloodmeals came from the same individual [63]. This finding demonstrates one of the major limitations of more quantitative tools based on host abundance (foraging ratio, etc), namely the inherent heterogeneity of sources for vector-feeding across populations of hosts [79]. This is an issue deserving more studies, taking advantage of molecular tools, like DNA fingerprinting [27,63,80]. This fact also leads to a possible mechanism linking host population size and feeding patterns, since larger populations have increased likelihoods of bearing stressed individuals [81], which are more likely to be bitten, primarily because of lack of defensive behaviors [77]. These same patterns also arise in neglected and minority populations or endangered species [81] which further supports the need to look qualitatively at presence/absence of hosts, since mosquito foraging may not necessarily be a functional response of host density [82].

From an applied perspective, and especially in the context of zoonotic vector-borne diseases, the broad range of blood hosts that may be fed upon by mosquitoes is one of the underpinnings for disease emergence, as was early recognized by the scientific community studying arboviral transmission [28,83,84] and has long been recognized as a regulatory factor for malaria transmission (see Additional file 2). In the mosquito communities we analyzed, most mosquito species can feed on at least one common host species independently of their innate preferences. Synchronization of encounters between hosts and vectors can promote disease emergence by facilitating foraging across a community of pathogen-susceptible hosts. Anthropogenic changes, such as deforestation and forest fragmentation can increase mosquito dispersal and foraging across abundant hosts [85], a pattern observed in other vectors such as sandflies [86] and ticks [87]. More generally, transmission control strategies such as insecticide treated nets that target host-vector contact have been the most successful at stopping the transmission of vector-borne diseases [88], demonstrating the importance of taking into account vector foraging [89]. From a theoretical perspective the explanation for the coupling of foraging specialization in organisms with niche shifts deserves further inquiry. The evolution of a life history strategy with different degrees of foraging specialization across ontogenetic stages could explain some cases of species co-occurrence and can be one mechanism for speciation in mosquitoes.

Finally, our study highlights the potential of mosquitoes as a model system to study foraging in organisms with niche shifts during their life cycle, where several factors are still unknown, such as the effects of nectar feeding on blood foraging and pathogen development [90]. It also illustrates the use of ecological theory and derived tools to further understanding of patterns that are of importance in studying and managing human diseases.

## Competing interests

The authors declare that they have no competing interests.

## Authors' contributions

LFC proposed the research idea, designed research, wrote an initial ms draft, and wrote additional files 1, 2 &4. LCH wrote additional file 3. Database search and study selection was done by CLK and AMN, with feedback from LFC, LCH & UDK. CLK prepared Tables 1 and 2. AMN prepared additional files 5, 6 &7. UDK helped to write additional file 2, provided comments on the spatial scale of studies. All authors performed research, contributed to the final draft, and have read and approved the manuscript.

## Supplementary Material

Additional file 1 **Biodiversity and zooprophylactic effects **[19,65,82,95-98]. Support information. Click here for file

Additional file 2 **Zooprophylaxis and dilution effect: the same natural history seen with different glasses **[95,99-104]. Support information. Click here for file

Additional file 3 **Mosquito blood-feeding analysis: from precipitin tests to DNA fingerprinting **[25,27,28,37,61,75,80,105-117]. Support information. Click here for file

Additional file 4 **A primer of null model testing of vector blood-feeding patterns **[34]. Support information. Click here for file

Additional file 5 **Table S1**. Mammal species considered in the bloodmeal identification. Percent of studies is relative to the number of papers that consider mammal blood meals. Click here for file

Additional file 6 **Table S2**. Bird species considered in the bloodmeal identification. Percent of studies is relative to the number of papers that consider Avian blood meals. Click here for file

Additional file 7 **Table S3**. Study sampling frequency and time length. Click here for file
