# Supplementary material for: Blood feeding patterns of mosquitoes: random or structured?
Source: Front Zool. 2010 Jan 21;7:3. doi: 10.1186/1742-9994-7-3 (PMC2826349; doi:10.1186/1742-9994-7-3)
Supplement: Additional file 1 — Biodiversity and zooprophylactic effects [19,65,82,95-98]. Support information. [file 1742-9994-7-3-S1.PDF]

Biodiversity and zooprophylactic effects

The mechanistic articulation of zooprophylaxis action was first modeled by Sota and Mogi [95] who confirmed that decreased transmission for a focal species was only feasible under a strong preference for bloodmeals from species other than the focal. In some instances an opposite outcome can be expected, known as zoopotentiation [96], when additional hosts can increase total transmission by increasing the abundance of vectors. Thus, if vector foraging on a given host species (β) is defined by the ratio between two functions, one for the effects of hosts on vector abundance (i.e., a[]) and one for the effects of host abundance on the foraging pressure by vectors on a specific host (i.e., f[]), then:

$$\beta = \frac{a[focal+others]}{f[focal+others]} \quad (1)$$

The zooprophylactic effect on transmission can be expected under three scenarios: (i) when vectors are limited by factors other than blood feeding, for example the abundance and quality of larval habitats [97], (ii) vectors preferentially feed on non-susceptible hosts [98], or (iii) when only a subset of the hosts can amplify the pathogen [19]. In all these cases the value of function a[] is expected to be a constant. However, in cases where either blood sources influence mosquito abundance, most blood-feeding hosts are susceptible to the pathogen, or vectors preferentially feed on the subgroup of hosts that are most susceptible to pathogen infection, the nonlinear relationships between both functions, together with spatial and behavioral factors favoring host-vector contact can explain the often conflicting results observed for zooprophylaxis, especially for malaria [65] or the preferential feeding on reservoir hosts over incidental hosts in other mosquito-borne diseases like Japanese Encephalitis [82]
