# Supplementary material for: Blood feeding patterns of mosquitoes: random or structured?
Source: Front Zool. 2010 Jan 21;7:3. doi: 10.1186/1742-9994-7-3 (PMC2826349; doi:10.1186/1742-9994-7-3)
Supplement: Additional file 2 — Zooprophylaxis and dilution effect: the same natural history seen with different glasses [95,99-104]. Support information. [file 1742-9994-7-3-S2.PDF]

Zooprophylaxis and dilution effect: the same natural history seen with different glasses

Over recent years, increasing interest among ecologists in the transmission of infectious diseases has given an impetus to the study of foraging behavior by bloodsucking arthropods, provided their central role in some pathogens' transmission. One of the observations derived from this scientific community has been the association between a decreased risk of infection with pathogens, especially vector-borne, and increased biodiversity, a trend nowadays defined as dilution effect [99]. However, this natural history observation is very old and can be traced back to the Historia Naturalis by Pliny the Elder who documented the emergence of malaria in areas of the ancient world where natural ecosystems were transformed by human action [100]. Although Pliny the Elder was wrong about changes in the air as causing malaria, at the beginning of the 20th century Angelo Celli, an Italian malariologist well aware of the role of mosquitoes on malaria transmission, realized that the association between deforestation and malaria that Pliny observed probably resulted from changes in mosquito foraging due to the loss of a diverse community of vertebrates as a consequence of forest clearance [100,101]. Celli proposed the term zooprophylaxis to define the control of malaria by the presence of alternative blood hosts [101]. Celli's hypothesis was supported by puzzling observations of malaria cases, *Anopheles* mosquitoes and absence of malaria transmission, named in the 1920's as anophelism without malaria [102]. This observation was explained when Hackett and Missiroli [103] showed increased foraging of mosquitoes on hosts other than humans in farmlands. However, the mainstream zooprophylaxis definition used by public health entomologists and infectious disease epidemiologists emerged from the assumption of preferential vector foraging on host species different from humans [95,104], ignoring the original reference to the more general effect on diluting pathogen transmission in a focal species of an increased diversity in the community of blood hosts.
