# Supplementary material for: Blood feeding patterns of mosquitoes: random or structured?
Source: Front Zool. 2010 Jan 21;7:3. doi: 10.1186/1742-9994-7-3 (PMC2826349; doi:10.1186/1742-9994-7-3)
Supplement: Additional file 3 — Mosquito blood-feeding analysis: from precipitin tests to DNA fingerprinting [25,27,28,37,61,75,80,105-117]. Support information. [file 1742-9994-7-3-S3.PDF]

Mosquito blood-feeding analysis: from precipitin tests to DNA fingerprinting

Identification of mosquito bloodmeals is fundamental for understanding mosquito foraging behavior and vector-borne disease ecology. Pioneering work on immunity and blood relationships by Nuttall in 1904 paved the way for the development of serological tests to differentiate between arthropod bloodmeals taken on different hosts. Nuttall [105] created several types of rabbit antisera that were tested to identify tick hosts [106]. The precipitin ring test was one of the first serological methods employed for identification of mosquito bloodmeals in the 1920's [25]. This test is named after the cloudy antibody-antigen complex that forms at the confluence of sera and antisera. Several variations of the precipitin test, including the agar gel diffusion and microplate tests were also developed [107, 108]. The precipitin ring test is highly dependent on the specificity and sensitivity of the antisera developed. This approach was used for many years [28, 37, 65] until the development of the enzyme-linked immunosorbent assay (ELISA) by Burkot et al. [109]. The sensitivity of the ELISA was improved with the antibody sandwich ELISA [110]. Unfortunately, only a small number of hosts can be identified with ELISAs due to limitations of commercially available reagents. ABO blood typing was developed and utilized for indentifying different human hosts of mosquitoes [75, 111]. Serious use of host DNA analysis was first employed by Coulson et al. [112] using repetitive segments in nuclear genes, aiming at identifying different human hosts of mosquitoes. The human DNA fingerprinting technique has been greatly improved with the use of microsatellite markers [27,80]. Microsatellites can be used for identification of a variety of different hosts within species such as individual birds [113]. In the past decade several new PCR-based approaches have been employed to identify mosquito bloodmeals. These newer methods have an advantage over the older precipitin- and ELISA-based methods. They are relatively simple to perform and can be much more sensitive. Unfortunately, they can be expensive especially considering the costs of sequencing, or when employing additional approaches such as restriction fragment length polymorphism (RFLP). With many DNA-based approaches, animal hosts of mosquitoes can be reliably identified to species level. These methods do have some drawbacks (reviewed by Kent et al. [114]). The most common approaches for bloodmeal analysis have employed mitochondrial genes, ribosomal RNA genes, and nuclear genes. Mitochondrial genes are present in multiple copies vastly increasing the amount of template available for amplification. Of the mitochondrial genes, *cytochrome b* (*cytb)* is the most commonly used [80]. One problem with mitochondrial genes is potential co-amplification of nuclear pseudo genes termed "numts", which can cause interpretation errors and have caused problems in phylogenetic studies [115]. PCR-RFLP using *cyt b* has been successfully employed for identification of tsetse blood hosts [116]. The *cytochrome c oxidase 1 (COI*) gene is another promising target for mosquito bloodmeal studies. One region of *COI* is the target of a large scale DNA-barcoding project (Barcode of Life Data Systems, www.barcodinglife.org. Preliminary data suggests that DNA barcoding works well for avian hosts (Lovette and Harrington, unpublished data). Ribosomal RNA genes have been used primarily for tick bloodmeals, but show some promise for identifying those of mosquitoes [117]. Molecular approaches for bloodmeal identification rely on matching sequences accurately to libraries which can create difficulties if posted data contain errors. As more numerous and accurate host sequence data become available, the specificity of DNA-based approaches to host bloodmeal identification will become increasingly powerful.
