# Supplementary material for: Blood feeding patterns of mosquitoes: random or structured?
Source: Front Zool. 2010 Jan 21;7:3. doi: 10.1186/1742-9994-7-3 (PMC2826349; doi:10.1186/1742-9994-7-3)
Supplement: Additional file 4 — A primer of null model testing of vector blood-feeding patterns [34]. Support information. [file 1742-9994-7-3-S4.PDF]

A primer of null model testing of vector blood-feeding patterns

Let's assume we have three communities of 5 mosquito species (rows) and 5 host species (columns). In the first community mosquitoes have a strong segregation in their feeding preferences that is realized in their choices, so that the emergent pattern from their host choices reflects their innate preferences. We would expect to see the following pattern (of perfect checkerboard):

1	0	1	0	1
0	1	0	1	0
1	0	1	0	1
0	1	0	1	0
1	0	1	0	1

The C- Score for this matrix is 3.5, which is larger than values obtained in 5000 out of a total of 5000 simulations (i.e., P=0.000), this pattern is known as **segregated** (or non-aggregated in the ecological literature [34]). In the second community mosquito species are not that choosy, and patterns are expected to have a lesser degree of segregation, yet they may show some non-overlapping host exploitation, like in the following matrix:

1	0	1	0	1
1	1	1	1	1
1	1	1	0	1
0	1	1	1	0
1	0	1	1	1

For this matrix the C- score is 0.9, which in 1076 out of 5000 simulations (21.5%) is equal to the simulated values, meaning that it is not statistically different from what is expected by random (P>0.05), indicating a **random** feeding pattern. The random pattern can be expected if host exploitation is unrelated to mosquito preferences, or by a degree of phenotypic plasticity where mosquitoes are choosy depending on host availability. In the third community all mosquito species primarily feed on a couple of species, resulting in a pattern like the following:

1	0	1	0	1
1	1	1	0	0
1	0	1	0	0
1	1	1	1	1
1	0	1	1	0

The C-score for this matrix is 0.3, which is smaller than the simulated scores in 4866 (97.32%) out of the 5000 simulations ($P<0.0268$), indicating that the feeding pattern is **aggregated**. This last pattern can be expected under at least two hypothetical scenarios, all mosquito species have the same innate preference for host species in a given community or most mosquito species select their hosts based on their defensive behavior or relative abundance in the community of vertebrates. In the examples presented here we used the fixed (rows)-equiprobable (columns) algorithm (see Methods).
