# Supplementary material for: Blood feeding patterns of mosquitoes: random or structured?
Source: Front Zool. 2010 Jan 21;7:3. doi: 10.1186/1742-9994-7-3 (PMC2826349; doi:10.1186/1742-9994-7-3)
Supplement: Additional file 5 — Table S1. Mammal species considered in the bloodmeal identification. Percent of studies is relative to the number of papers that consider mammal blood meals. [file 1742-9994-7-3-S5.PDF]

Table S1 Mammal species considered in the bloodmeal identification. Percent of studies is relative to the number of papers that consider mammal blood meals.

Species	Percent of studies	Papers
Unspecified Mammal	50.00	(Apperson *et al.* 2002; Edman 1971; Edman 1979; Nasci & Edman 1981)
Armadillo	12.50	(Edman 1971; Edman 1979)
Flying Fox	6.25	(Kay et al. 2007)
Cat	50.00	(Apperson *et al.* 2004; Fyodorova *et al.* 2006; Hamer 2009; Hess & Hayes 1970; Kay *et al.* 2007; Molaei *et al.* 2008; Savage *et al.* 2007)
Chipmunk	6.25	(Molaei et al. 2008)
Dog	75.00	(Apperson *et al.* 2002; Apperson *et al.* 2004; Fyodorova *et al.* 2006; Hamer 2009; Kay *et al.* 2007; Molaei *et al.* 2008; Nasci & Edman 1981; Savage *et al.* 2007)
Fox	6.25	(Molaei et al. 2008)
Horse	68.75	(Apperson *et al.* 2004; Forattini *et al.* 1989; Fyodorova *et al.* 2006; Kay *et al.* 2007; Molaei *et al.* 2008; Nasci & Edman 1981; Savage *et al.* 2007)
Human	75.00	(Apperson *et al.* 2002; Apperson *et al.* 2004; Forattini *et al.* 1989; Fyodorova *et al.* 2006; Hamer 2009; Kay *et al.* 2007; Molaei *et al.* 2008; Nasci & Edman 1981; Savage *et al.* 2007)
Macropod	6.25	(Kay et al. 2007)
Marsupial	6.25	(Forattini et al. 1989)
Opossum	50.00	(Apperson *et al.* 2004; Edman 1971; Edman 1979; Hamer 2009; Kay *et al.* 2007; Molaei *et al.* 2008; Savage *et al.* 2007)
Otter	6.25	(Molaei et al. 2008)
Pig	31.25	(Apperson *et al.* 2004; Fyodorova *et al.* 2006; Nasci & Edman 1981)
Rabbit	81.25	(Apperson *et al.* 2002; Apperson *et al.* 2004; Edman 1971; Edman 1979; Fyodorova *et al.* 2006; Hamer 2009; Molaei *et al.* 2008; Nasci & Edman 1981; Savage *et al.* 2007)
Raccoon	43.75	(Apperson *et al.* 2002; Apperson *et al.* 2004; Edman 1971; Edman 1979; Hamer 2009; Molaei *et al.* 2008; Savage *et al.* 2007)
Unspec. Rodent	37.50	(Apperson *et al.* 2002; Edman 1979; Forattini *et al.* 1989; Nasci & Edman 1981)
Rats	12.50	(Molaei *et al.* 2008)
Unspec. Ruminant	12.50	(Edman 1971; Edman 1979)
Bovine	50.00	(Forattini *et al.* 1989; Fyodorova *et al.* 2006; Molaei *et al.* 2008; Nasci & Edman 1981)
Deer	31.25	(Apperson *et al.* 2004; Hamer 2009; Molaei *et al.* 2008; Savage *et al.* 2007)
Squirrel	25.00	(Apperson *et al.* 2004; Hamer 2009; Molaei *et al.* 2008; Savage *et al.* 2007)
