# Supplementary material for: Blood feeding patterns of mosquitoes: random or structured?
Source: Front Zool. 2010 Jan 21;7:3. doi: 10.1186/1742-9994-7-3 (PMC2826349; doi:10.1186/1742-9994-7-3)
Supplement: Additional file 6 — Table S2. Bird species considered in the bloodmeal identification. Percent of studies is relative to the number of papers that consider Avian blood meals. [file 1742-9994-7-3-S6.PDF]

Table S2 Bird species considered in the bloodmeal identification. Percent of studies is relative to the number of papers that consider Avian blood meals.

Avian species	% of papers	Papers
Acadian flycatcher	16.67	(Molaei et al. 2008)
Amerian Kestrel	33.33	(Savage et al. 2007), (Hamer 2009)
American crow	50.00	(Apperson *et al.* 2004; Molaei *et al.* 2006; Savage *et al.* 2007)
American robin	100.00	(Apperson *et al.* 2002; Apperson *et al.* 2004; Hamer 2009; Molaei *et al.* 2006; Molaei *et al.* 2008; Savage *et al.* 2007)
American_goldfinch	16.67	(Hamer 2009)
American woodcock	16.67	(Molaei et al. 2008)
Barn swallow	66.67	(Apperson *et al.* 2002; Apperson *et al.* 2004; Molaei *et al.* 2006; Savage *et al.* 2007)
Black-and-white warbler	16.67	(Molaei et al. 2006)
Black-capped chickadee	33.33	(Hamer 2009; Molaei *et al.* 2006)
Black-crowned night heron	16.67	(Molaei et al. 2006)
Blue Jay	50.00	(Apperson *et al.* 2004; Hamer 2009; Savage *et al.* 2007)
Blue-headed vireo	16.67	(Molaei et al. 2006)
Brown Thrasher	50.00	(Apperson *et al.* 2002; Hamer 2009; Savage *et al.* 2007)
Brown-headed cowbird	83.33	(Apperson *et al.* 2002; Apperson *et al.* 2004; Hamer 2009; Molaei *et al.* 2006; Savage *et al.* 2007)
Canada goose	16.67	(Molaei et al. 2006)
Carolina chickadee	33.33	(Apperson *et al.* 2002; Apperson *et al.* 2004)
Cedar waxwing	66.67	(Apperson *et al.* 2002; Hamer 2009; Molaei *et al.* 2006; Savage *et al.* 2007)
Chicken	33.33	(Hamer 2009; Savage *et al.* 2007)
Chipping_sparrow	16.67	(Hamer 2009)
Common grackle	83.33	(Apperson *et al.* 2002; Hamer 2009; Molaei *et al.* 2006; Molaei *et al.* 2008; Savage *et al.* 2007)
Common Yellowthroat	16.67	(Savage et al. 2007)
Common_canary	16.67	(Hamer 2009)
Coopers_hawk	16.67	(Hamer 2009)
Eastern_blulebird	16.67	(Hamer 2009)
Eastern_towhee	16.67	(Hamer 2009)

European starling	66.67	(Hamer 2009; Molaei *et al.* 2006; Molaei *et al.* 2008; Savage *et al.* 2007)
Field Sparrow	66.67	(Apperson *et al.* 2002; Apperson *et al.* 2004; Hamer 2009; Savage *et al.* 2007)
Green heron	33.33	(Molaei *et al.* 2006; Savage *et al.* 2007)
Green-backed heron	16.67	(Apperson et al. 2004)
Grey catbird	83.33	(Apperson *et al.* 2004; Hamer 2009; Molaei *et al.* 2006; Molaei *et al.* 2008; Savage *et al.* 2007)
Hermit thrush	16.67	(Molaei et al. 2008)
House finch	66.67	(Hamer 2009; Molaei *et al.* 2006; Molaei *et al.* 2008; Savage *et al.* 2007)
House sparrow	66.67	(Apperson *et al.* 2004; Hamer 2009; Molaei *et al.* 2006; Savage *et al.* 2007)
House wren	33.33	(Hamer 2009; Molaei *et al.* 2006)
Indigo bunting	16.67	(Molaei et al. 2006)
Mallard	33.33	(Hamer 2009; Molaei *et al.* 2006)
Mixed Avian	33.33	(Apperson *et al.* 2002; Savage *et al.* 2007)
Mixed norhtern cardinal	16.67	(Apperson et al. 2004)
Mourning dove	66.67	(Hamer 2009; Molaei *et al.* 2006; Molaei *et al.* 2008; Savage *et al.* 2007)
Northern cardinal	83.33	(Apperson *et al.* 2002; Apperson *et al.* 2004; Hamer 2009; Molaei *et al.* 2006; Savage *et al.* 2007)
Northern Mockingbird	50.00	(Apperson *et al.* 2002; Apperson *et al.* 2004; Savage *et al.* 2007)
Northern oriole	16.67	(Molaei et al. 2006)
Northern waterthrush	16.67	(Molaei et al. 2006)
Northern_flicker	16.67	(Hamer 2009)
Pine warbler	16.67	(Apperson et al. 2004)
Prairie warbler	16.67	(Molaei et al. 2006)
Red Winged Blackbird	33.33	(Apperson *et al.* 2004; Savage *et al.* 2007)
Red-tailed hawk	16.67	(Molaei et al. 2006)
Red-winged blackbird	33.33	(Hamer 2009; Molaei *et al.* 2006)
Ring-necked pheasant	33.33	(Apperson *et al.* 2002; Apperson *et al.* 2004)
Rock dove	16.67	(Molaei et al. 2006)
Rock_pigeon	16.67	(Hamer 2009)
Rose-brested grosbeak	16.67	(Molaei et al. 2006)
Scarlet tanager	50.00	(Apperson *et al.* 2004; Hamer 2009; Molaei *et al.* 2008)

Sharp-shinned hawk	16.67	(Molaei et al. 2006)
Song sparrow	33.33	(Hamer 2009; Molaei *et al.* 2006)
Swainsons_thrush	16.67	(Hamer 2009)
Swamp_sparrow	16.67	(Hamer 2009)
Tufted Titmouse	33.33	(Apperson *et al.* 2004; Savage *et al.* 2007)
Unidentifiable	33.33	(Apperson *et al.* 2002; Apperson *et al.* 2004)
Veery	16.67	(Hamer 2009)
Wild turkey	50.00	(Hamer 2009; Molaei *et al.* 2006; Molaei *et al.* 2008)
Willow flycatcher	16.67	(Molaei et al. 2006)
Wood duck	16.67	(Molaei et al. 2006)
Wood thrush	50.00	(Molaei *et al.* 2006; Molaei *et al.* 2008)
Yellow-billed Cuckoo	16.67	(Savage et al. 2007)
