# Supplementary material for: Blood feeding patterns of mosquitoes: random or structured?
Source: Front Zool. 2010 Jan 21;7:3. doi: 10.1186/1742-9994-7-3 (PMC2826349; doi:10.1186/1742-9994-7-3)
Supplement: Additional file 7 — Table S3. Study sampling frequency and time length. [file 1742-9994-7-3-S7.PDF]

Table S3 Study sampling frequency and time length

Study	Study sampling frequency and time length
**Florida** Edman(1971, 1974a, 1974b, 1979); Edman et al (1972) ; Edman and Hager (1977)	1 weekly sample during June and July from 1966 to 1970 in Edman (1971, 1974a, 1974b). Variable in the other studies
**Connecticut** Molaei et al (2006, 2008)	No information on sampling frequency. From June to October 2002-2007 (Molaei et al 2008). From June to October 2002-2004 (Molaei et al 2006)
Apperson et al (2002)	2 weeks (July and August) 2000
Nasci and Edman (1981)	14 nights during the summer of 1978
Burkett-Cadena et al (2008)	1 weekly sample from February to October 2007
Forattini et al (1989)	Monthly from February to November 1986
Apperson et al (2004)	No information on sampling frequency. Sampling between May and October in New Jersey, 2001; and between June and September in New York, 2001.
Savage et al (2007)	At least monthly from February 2002 to December 2003.
Hamer et al (2009)	Biweekly from mid-May to mid-October during 2005 to 2007
Kay et al (2001)	Twice weekly from September 2000 to April 2001
Fyodorova et al (2006)	Sites were sampled once during two sampling intervals in 2003
